# General anesthetic management for dental surgery in an adult patient with Rubinstein-Taybi syndrome

**DOI:** 10.1186/s40981-023-00621-z

**Published:** 2023-05-20

**Authors:** Atsushi Kotera

**Affiliations:** Department of Anesthesiology, Kumamoto City Hospital, 4-1-60, Higashimachi, Higashi-Ku, Kumamoto City, 862-8505 Japan

To the Editor

Rubinstein-Taybi syndrome (RTS) is a rare genetic disorder [1], and the incidence is approximately 1/100,000 [2]. Its most notable feature is facial dysmorphism, e.g., widely spaced eyes, a beak-shaped nose, and micrognathia [3], and difficult airway management is challenging [4]. I present the general anesthetic management for dental surgery in a patient with RTS.

A 31-year-old female (body weight, 50 kg; height, 139 cm) was scheduled for dental surgery under general anesthesia. She was diagnosed with RTS at birth and presented typical characteristic for RTS, including mental retardation, short stature, widely spaced eyes, a beak-shaped nose, and broad thumbs (Fig. 1A, B). She was hospitalized for aspiration pneumonia at 26 and 28 years, and severe carious teeth were detected. Her respiratory condition was normal with peripheral oxygen saturation of 96% in room air. Cervical X-ray showed no choanal atresia (Fig. 1C), but brain CT demonstrated a nasal septum deviation (Fig. 1D).

**Fig. 1:**
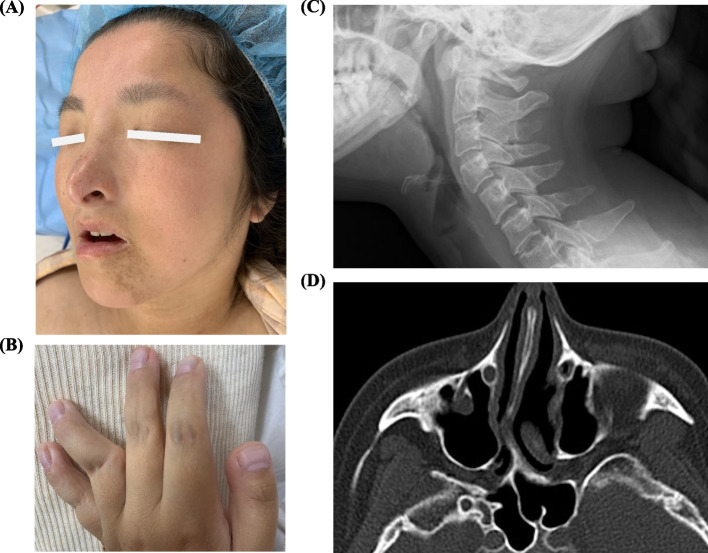
Surface appearance and radiographic images of the patient with Rubinstein-Taybi syndrome. **A** Slanting, widely spaced eyes and a beak-shaped nose but not remarkable micrognathia. **B** A broad thumb and a bended ring finger in the left hand. **C** Non-choanal atresia and airway narrowing in cervical X-ray. **D** Remarkably deviated nasal septum in brain CT

Oral premedication with 10 mg diazepam was administered. General anesthesia was inducted with 3 mg midazolam, 50 μg fentanyl, and 40 mg propofol in the reverse Trendelenburg position. Face mask ventilation was confirmed to be possible before administering 30 mg rocuronium. Oral intubation was tried with a conventional laryngoscope. Tonsillar hypertrophy was observed, but the laryngoscopic view was defined as Cormack and Lehane grade II-b by using the Burp maneuver, and an ID 6.5-mm cuffed tracheal tube was inserted. Both nasal cavities were disinfected carefully, and an ID 6.0-mm cuffed tracheal tube was inserted through the left nostril with a video laryngoscope. General anesthesia was maintained with 4–5 mg/kg/h propofol and 0.15–0.2 μg/kg/min remifentanil, and 6 μg/kg fentanyl was administered. Her intraoperative respiratory condition was stable with peripheral oxygen saturation of 98% on 35% oxygen. For antiemesis, 6.6 mg dexamethasone and 4 mg ondansetron were given. The surgical duration was 149 min, and the tracheal tube was removed after confirming that the ratio of train-of-four showed > 0.9, and she was discharged on the next day uneventfully.

In this present case, nasal intubation was predicted to be difficult because of a nasal septum deviation, but oral intubation was thought to be enforceable because of unremarkable micrognathia. Therefore, oral intubation prior to nasal intubation was selected. That was noted in a pediatric patient with RTS [5], and it insured enough time for disinfection of both nasal cavities, confirmation of the passage of each nasal cavity, and decision of tracheal tube size. Furthermore, nasal trauma could be avoided.

In a patient with RTS, a recurrent respiratory infection following dysphagia or gastroesophageal reflux is also remarkable. Attention should thus be paid to reduce the risk of aspiration or vomiting. In this present case, the reverse Trendelenburg position during the induction of general anesthesia, selection of intravenous anesthetics, and prophylactic administration of antiemetics were undergone. But, as in the other literatures [5–7], H_2_-antagonist should have been administered as premedication.

In conclusion, in a patient with RTS, careful anesthetic planning is necessary to achieve successful airway management and to prevent respiratory complications.

## Data Availability

The datasets for the reported patient are available from the corresponding author on reasonable request.

## Abbreviation

RTS: Rubinstein-Taybi syndrome
